# USABILITY OF A TABLET-BASED HOME MEMORY TEST IN OLDER ADULTS WITH AND WITHOUT COGNITIVE IMPAIRMENT

**DOI:** 10.1093/geroni/igad104.2762

**Published:** 2023-12-21

**Authors:** Yanchen Liu, Lisbeth Sanders, Igor Vishnepolskiy, Rebecca Williams, Jeff Sanders, Anthony Serpico, Stefan Gravenstein, Thomas Bayer

**Affiliations:** Brown University, Providence, Rhode Island, United States; LifeBio, Marysville, Ohio, United States; Lifespan, Providence, Rhode Island, United States; LifeBio, Marysville, Ohio, United States; LifeBio, Marysville, Ohio, United States; LifeBio, Marysville, Ohio, United States; Brown University, Providence, Rhode Island, United States; Brown University, Providence, Rhode Island, United States

## Abstract

Increasing Alzheimer’s and related disorders prevalence, highlights the need for accurate and reliable cognitive screening and diagnostic tools. We studied usability of LifeBio Brain, a prototype cognitive testing mobile application. We established baseline cognitive performance in volunteers from Brown University’s Geriatric Clinic using the Saint Louis University Mental Status (SLUMS). We asked volunteers to use the LifeBio Brain app at home on an iPad twice daily for 10 times, for about 15 minutes each time, followed by completion of a 10-question System Usability Scale on satisfaction that assesses the app and its ease of use. The LifeBio Brain app contains a mix of adapted cognitive tests such as clock draw and Trails A, and game-like experiences, presented in a randomized order. We analyzed a white non-hispanic sample (n=51) with a balanced distribution of age [79.6 (7.2) years, range 66-95] and sex (58.82% women). The median SLUMS score of 25, indicates cognitive impairment in most participants. Similarly, the median usability score on the 50-point System Usability Scale was 41. We found that 80% of participants had a usability score of 35 or greater, with a 95% CI of 31-38, and 67% had a usability score of 40 or greater. SLUMS score ≤14 correctly predicted study completion in 90% of participants. Most volunteers from an outpatient geriatrics clinic found LifeBio Brain usable. Volunteers leaving the study early tended to have lower SLUMS Scores. The LifeBio Brain app may have utility for measuring memory and thinking abilities in a white outpatient geriatrics population.

